# Sub-apoptotic dosages of pro-oxidant vitamin cocktails sensitize human melanoma cells to NK cell lysis

**DOI:** 10.18632/oncotarget.5024

**Published:** 2015-09-05

**Authors:** Elisa Tremante, Lory Santarelli, Elisa Lo Monaco, Camilla Sampaoli, Tiziano Ingegnere, Roberto Guerrieri, Marco Tomasetti, Patrizio Giacomini

**Affiliations:** ^1^ Laboratory of Immunology, Regina Elena National Cancer Institute, 00144 Rome, Italy; ^2^ Department of Clinical and Molecular Sciences, Polytechnic University of Marche, 60020 Ancona, Italy; ^3^ Center of Excellence on Electronic Systems (ARCES), University of Bologna, 40123 Bologna, Italy

**Keywords:** melanoma, autoschizis, oxidative stress, NKG2D, Natural Cytotoxicity Receptors (NCR)

## Abstract

Alpha-tochopheryl succinate (αTOS), vitamin K3 (VK3) and vitamin C (ascorbic acid, AA) were previously shown to synergistically promote different death pathways in carcinoma cells, depending on their concentrations and combinations. Similar effects were observed herein in melanoma cells, although αTOS behaved as an antagonist. Interestingly, suboptimal cell death-inducing concentrations (1.5 μM αTOS/20 μM AA/0.2 μM VK3) effectively up-regulated activating Natural Killer (NK) cell ligands, including MICA (the stress-signaling ligand of the NKG2D receptor), and/or the ligands of at least one of the natural cytotoxicity receptors (NKp30, NKp44 and NKp46) in 5/6 melanoma cell lines. Only an isolated MICA down-regulation was seen. HLA class I, HLA class II, ULBP1, ULBP2, ULBP3, Nectin-2, and PVR displayed little, if any, change in expression. Ligand up-regulation resulted in improved lysis by polyclonal NK cells armed with the corresponding activating receptors. These results provide the first evidence for concerted induction of cell death by cell-autonomous and extrinsic (immune) mechanisms. Alarming the immune system much below the cell damage threshold may have evolved as a sensitive readout of neoplastic transformation and oxidative stress. Cocktails of vitamin analogues at slightly supra-physiological dosages may find application as mild complements of melanoma treatment, and in chemoprevention.

## INTRODUCTION

The triad apoptosis, autophagy, and necrosis recapitulates the major accepted pathways of cell death [1]. Alpha-tochopheryl succinate (αTOS, a redox-silent analogue of vitamin E), vitamin K3 (VK3) and vitamin C (ascorbic acid, AA) induce apoptotic cell death through oxidative stress and mitochondrial destabilization. Of interest, these compounds retain biological activity even at subapoptotic concentrations. For instance, VK3 and AA induce a caspase 3-independent cell death termed autoschizis [2, 3], known to be intermediate between apoptosis and necrosis [4]. Death-inducing dosages may be further lowered by combining αTOS, VK3 and AA in a ‘cocktail’ [5]. Thus, changing the concentrations and combinations of αTOS, VK3 and AA is a convenient pharmacological strategy to tune oxidative stress, and selectively trigger distinct pathways of cell death.

One may then hypothesize that the αTOS/VK3/AA cocktail might be effective even below its death-induction threshold. At these ultra-low dosages, the cocktail may no longer induce direct cell death, but may trigger systemic sensors of cellular stress such as Natural Killer (NK) cells, that are programmed to recognize and kill target cells expressing stress ligands [6–9].

Immune ligands that directly determine lytic outcomes include: (a) classical Major Histocompatibility Complex class I molecules (MHC I), that are both activating and inhibitory; (b) non-classical MHC I molecules such as the activating MHC I-Related Chain A (MICA), UL40 Binding Protein (ULBP) 1, ULBP2 and ULBP3; and (c) non-MHC ligands such as Nectin 2 (CD112) and PolyoVirus Receptor (PVR, CD155). MICA and ULBPs bind the triggering receptor NKG2D; Nectin 2 and PVR bind DNAX accessory molecule 1 (DNAM-1; CD226). Other activating ligands, mostly unknown, bind the Natural Cytototoxicity Receptors (NCRs) NKp30, NKp44 and NKp46 [10, 11]. To our knowledge, none of these ligand:receptor systems has been implicated in pro-oxidant treatment.

A combination of cDNA arrays, immunohistochemistry, serology and immunological methods recently identified a combined overexpression signature of MICA, ULBPs and PVR in early-passage melanoma cells as compared to autologous normal melanocytes from the surrounding, intact skin of melanoma patients [12]. On this basis, a panel comprising continuous and early-passage melanoma cells was selected for treatment at sub-apoptosis and sub-autoschizis dosages with a cocktail containing αTOS, VK3 and AA. These cells were assessed for the expression of immune ligands and their susceptibility to NK cell lysis.

To our knowledge, this is the first study addressing the existence of a direct link between two different, and possibly convergent, programs leading to tumor cell death: the classical, hard-wired, high-dose program sustained by cell-autonomous mechanisms, and a novel putative program, preliminarily described herein, that is low-dose, soft-wired, extrinsic, and electively immune in nature.

## RESULTS

### Cell death induction by αTOS, AA and VK3 in melanoma: Dose-dependence and mechanisms

**Figure 1:**
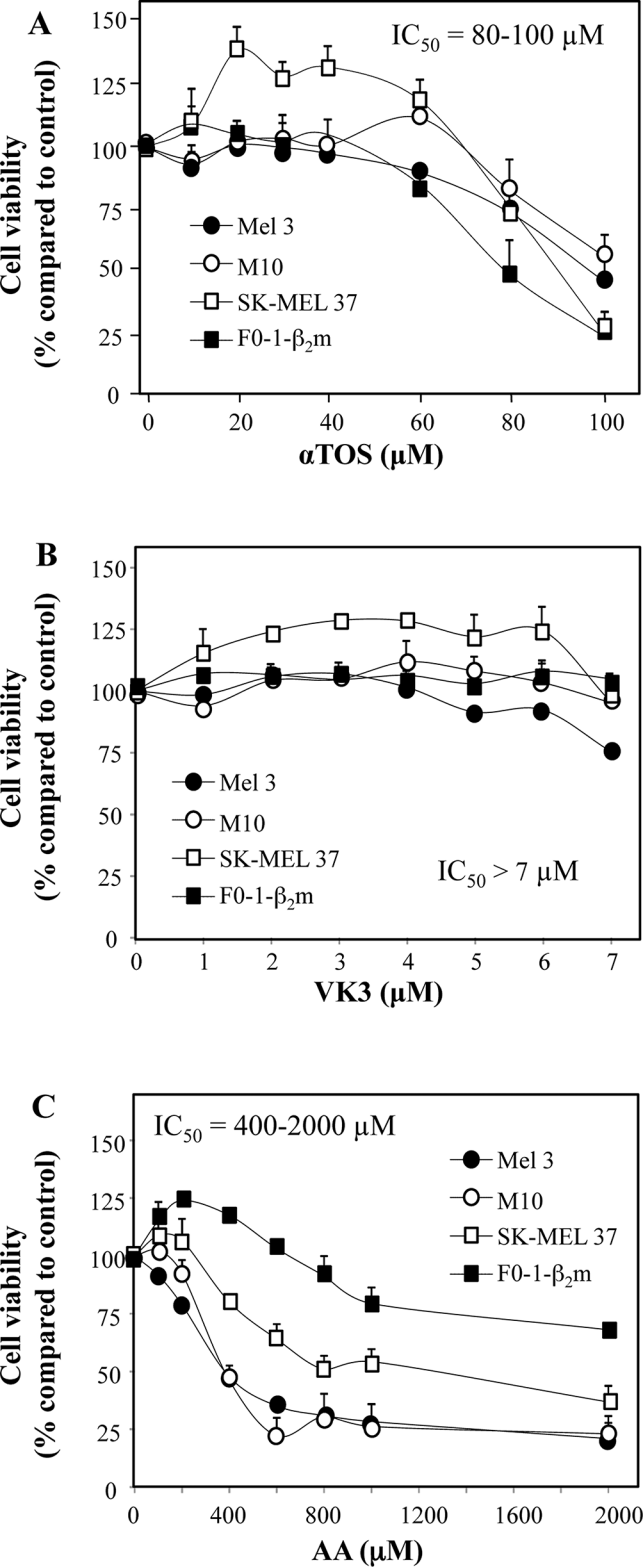
Induction of cell death in melanoma cell lines treated with αTOS, AA and VK3 The indicated cell lines were seeded into 96-well tissue culture plates at 3 × 10^4^ per well, and were treated for 24 h with increasing concentration of αTOS, VK3, and AA. Their viability was assessed by crystal violet.

In previous studies, some of us reported that αTOS, AA and VK3 act both individually and synergistically to induce prostate carcinoma cell death [5, 13]. In partial analogy with prostate carcinoma, single-agent treatment with αTOS and AA induced death in 4 melanoma cell lines at 80–100 μM and 400–2000 μM respectively, whereas VK3 had a negligible effect over a wide range of concentrations (Fig. 1).

**Figure 2:**
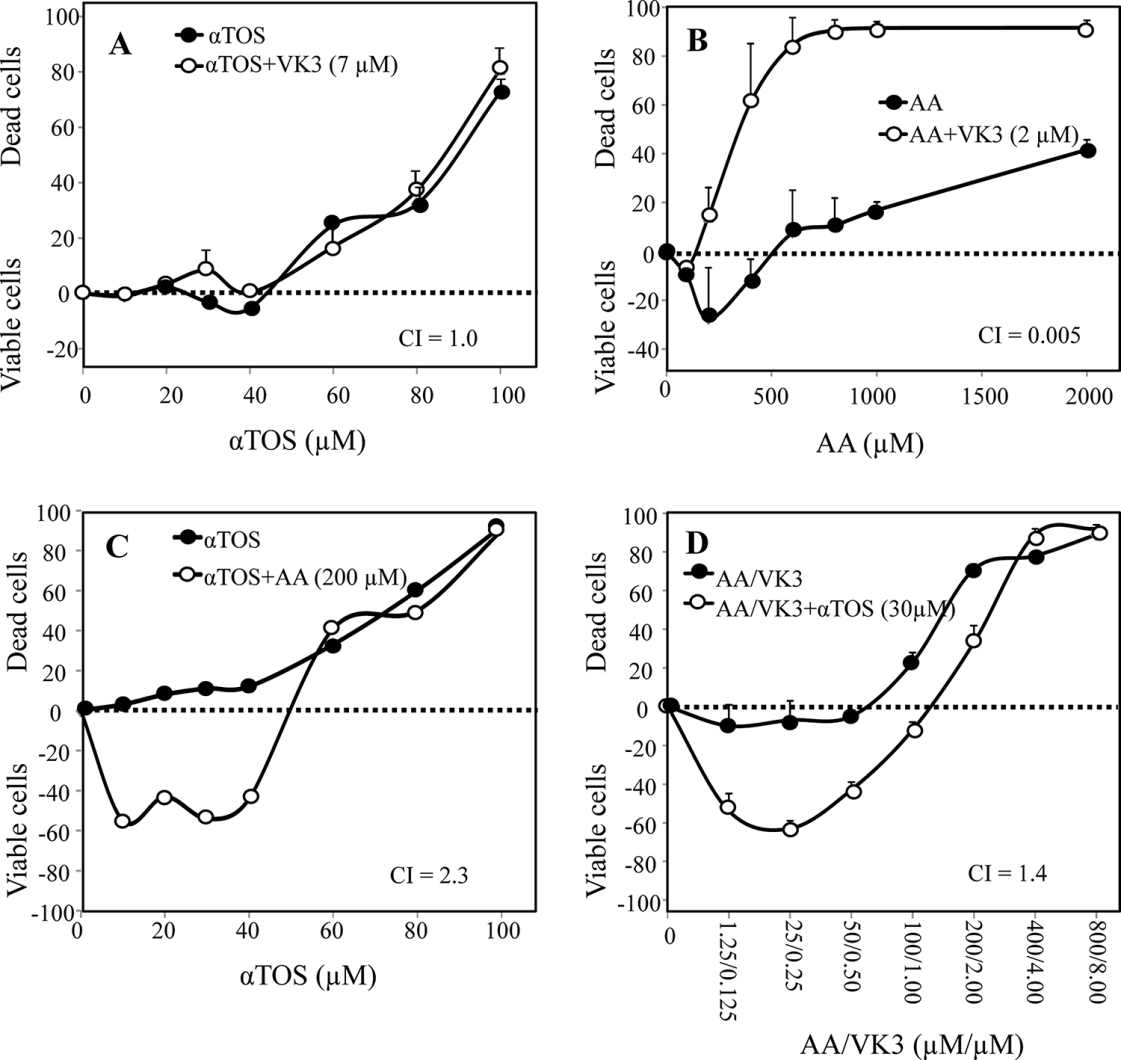
Synergistic induction of cell death with αTOS, AA and VK3 F0-1-β_2_m melanoma cells were treated with αTOS, AA and VK3 (alone and in combination) at the indicated concentrations. Cell death was assessed by crystal violet, and was expressed in the form of a Combination Index (CI), calculated as described in Materials and Methods. Synergistic (CI < 1), additive (CI = 1) or antagonistic (CI > 1) effects of sets of two **A–C.** and three **D.** drugs are expressed as the mean ± SD of the percentage variation with respect to the control (untreated cells) of 3 independent experiments carried out in duplicate.

Next, synergistic interactions were assessed in a representative melanoma cell line (F0-1-β_2_m) treated with αTOS and/or AA and/or VK3. VK3 remained essentially ineffective in combination with αTOS (Fig. 2A), but it did synergize with AA (Fig. 2B). In contrast, and surprisingly, αTOS did not increase, but rather suppressed, cell death induced by either AA alone (Fig. 2C) or the optimal VK3/AA combination (Fig. 2D).

**Figure 3:**
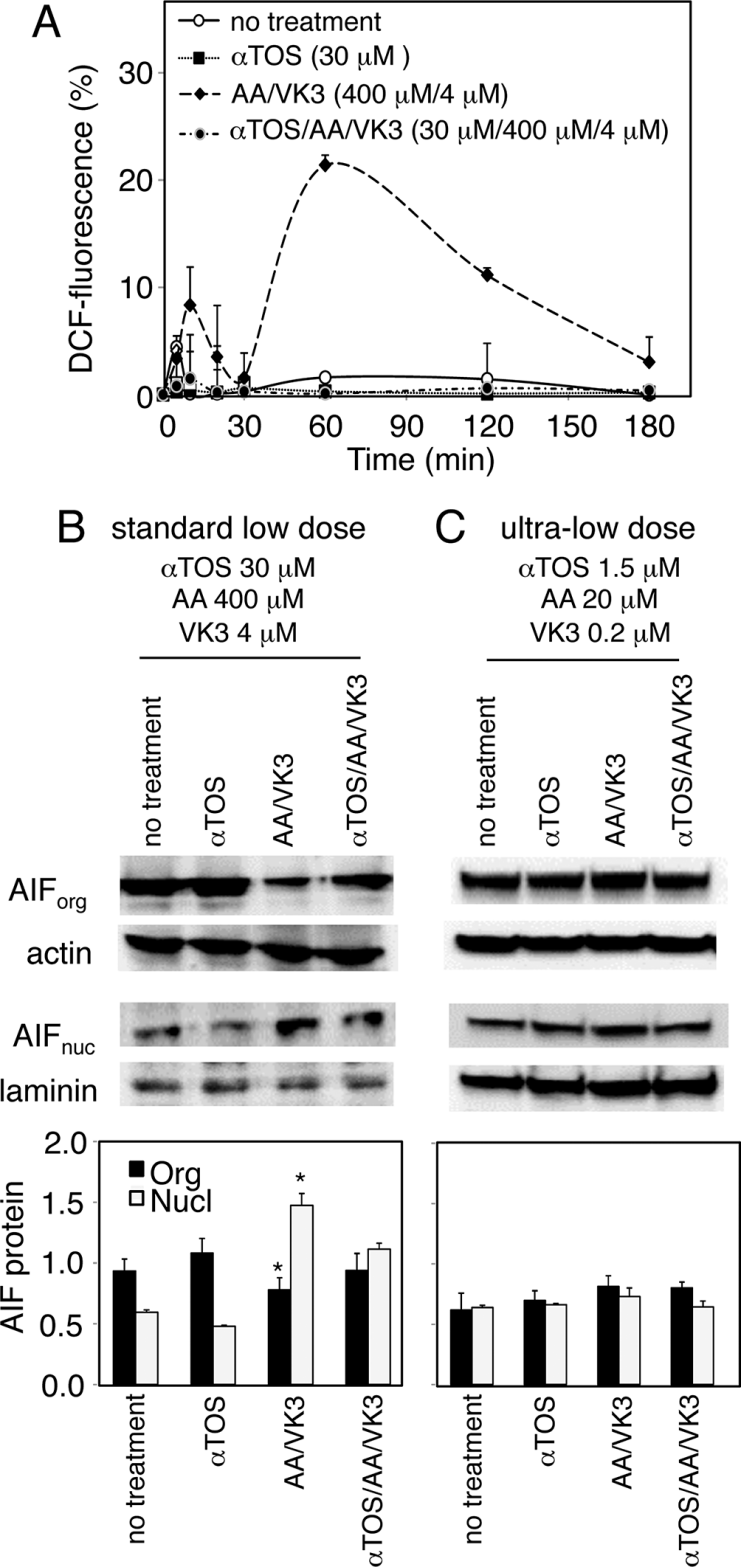
Effect of αTOS on the formation of Reactive Oxygen Species (ROS) and the nuclear translocation of Apoptosis Inducing Factor (AIF) **A.** F0-1-β_2_m cells were treated as indicated, and ROS were assessed in a time course experiment by measuring the oxidation of 2′7′-dichlorofluorescin diacetate to 2′7′-dichlorofluorescein (DCF). **B** and **C.** F0-1-β_2_m cells were treated for 180 minutes as indicated, and AIF was assessed in organelle (org), and nuclear (nucl) fractions by Western blotting. Densitometric values of specific bands was normalized to actin (AIForg) and laminin (AIFnucl), and displayed as mean values ±SD of three independent experiments. Comparisons among groups were determined by one-way ANOVA with Tukey post-hoc analysis; asterisks mark significant differences between treated and untreated cells (*p* < 0.05).

To understand why αTOS acts as a pure agonist in prostate carcinoma but behaves as an antagonist in melanoma, we assessed Reactive Oxygen Species (ROS) and nuclear translocation of the Apoptosis Inducing Factor (AIF). The latter is a crucial cell death induction checkpoint in prostate carcinoma [5, 13]. F0-1-β_2_m melanoma cells were treated with αTOS alone, AA/VK3, or αTOS/AA/VK3 at concentrations (30 μM αTOS, 400 μM AA, and 4 μM VK3; standard low dose hereafter) that efficiently induce cell death upon combination treatment (see Fig. 2D). Marked antagonistic effects of αTOS were observed on both ROS formation and AIF translocation induced by the AA/VK3 combination (Fig. 3A and 3B). These observations account for the peculiar antagonistic effects of αTOS in melanoma.

However, as shown in Fig. 3C, agonistic (AA/VK3) as well as antagonistic (αTOS) effects were no longer appreciable when the cocktail was diluted 20-fold (1.5 μM αTOS, 20 μM AA, 0.2 μM VK3; ultra-low dosage hereafter). In summary, αTOS does not appreciably interfere with AA and VK3 at ultra-low cocktail dosages, e.g. when concentration drops below a critical death-inducing threshold.

### Identification of a subliminal death-inducing dosage of the αTOS/AA/VK3 cocktail

**Figure 4:**
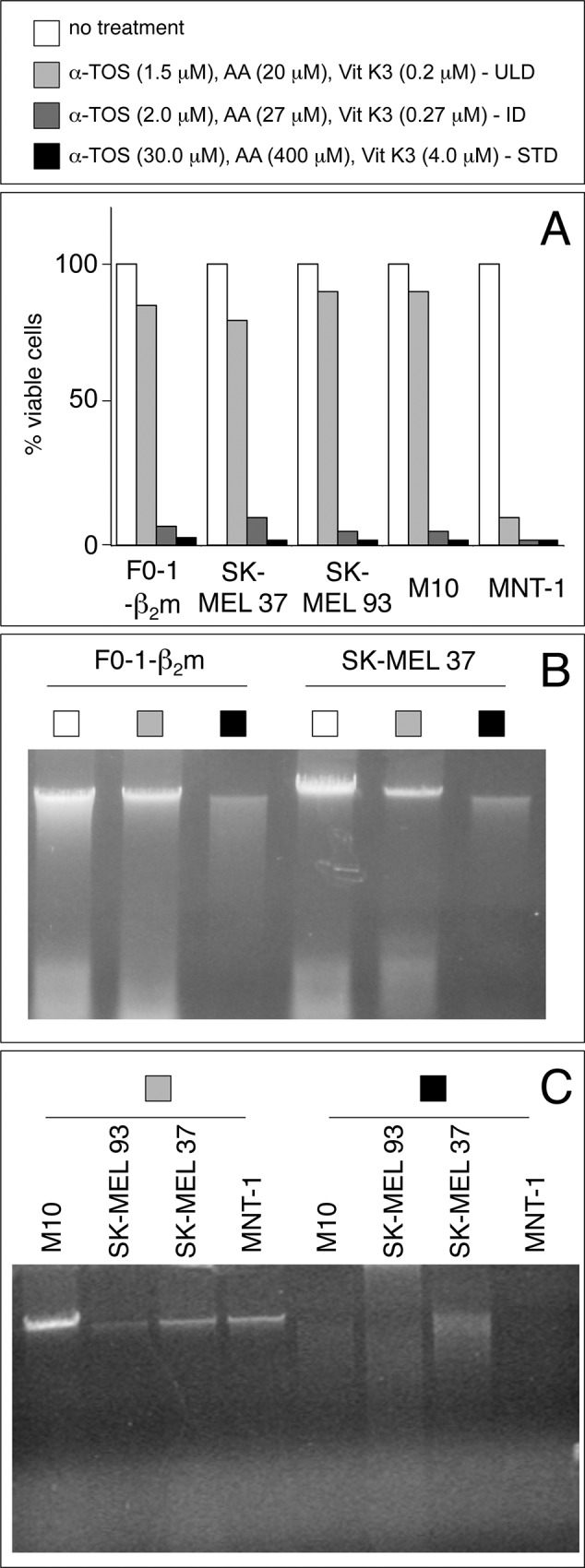
Identification of a subliminal death-inducing dosage of the αTOS/AA/VK3 cocktail **A.** melanoma cell lines were treated for 16 h at the indicated dosages of the cocktail (refer to the top panel for bar colors), and assessed for propidium iodide exclusion (% of viable cells) in a flow cytometer (Becton & Dickinson, Mountain View, CA). **B** and **C.** genomic DNAs from cells treated as in A at the indicated (refer to top panel) concentrations were electrophoresed under native conditions and visualized by EtBr staining. ULD, Ultra-Low Dosage; ID, intermediate Dosage; STD, Standard Dosage.

Based on the above results, F0-1-β_2_m and 9 additional melanoma cell lines were tested at three dosages: the standard low dosage, its 20-fold dilution (ultra-low dosage), and an intermediate 15-fold dilution. All the tested cells were sensitive to the cocktail, but to different extents, as shown by propidium iodide uptake in 5 representative cell lines (Fig. 4A). Four continuous melanoma cell lines (F0-1-β_2_m, SK-MEL 37, SK-MEL 93 and M10) were the least sensitive. They were efficiently killed at both the standard low dosage and at the intermediate 15-fold dilution, but a slight further dilution (20-fold, coinciding with the ultra-low dosage) resulted in a sharp, partial recovery in cell viability, with propidium iodide uptake decreasing below 20% (Fig. 4A). A similar recovery was seen in 2 early-passage cell lines (Mel 11 and Mel 24; not shown), but not in another patient-derived cell line (MNT-1), that remained extremely sensitive even at the ultra-low cocktail dosage, propidium iodide uptake exceeding 90% at all dosages (Fig. 4A). Finally, the remaining early-passage cell lines (Mel 3, Mel 23, and Mel 35) were the most sensitive, in that they displayed > 80% propidium iodide uptake even at dosages 5 times lower than the ultra-low dosage (not shown). As expected, the typical autoschizis genomic DNA smearing was exclusively visible at the standard low dosage in the resistant, continuous cell lines (representative results in Fig. 4B and 4C).

In summary, the αTOS/AA/VK3 cocktail induces overt cell death in 10 melanoma cells, but 6 of them display a sharp cytotoxic threshold between the intermediate and the ultra-low dosages. These 6 cell lines were selected to assess changes in the expression of immune ligands at subliminal (right below threshold) death-inducing regimens. MNT-1 cells were included as a control, whereas the remaining early-passage cell lines were not further tested in light of their extreme sensitivity to the cocktail and lack of a threshold effect in the selected dosage range.

### Immunophenotypic up-regulation of activating NK cell ligands

MHC class I (HLA-A, -B, -C in humans) and MHC class II (HLA-DR, -DQ, -DP) molecules were tested first. We found that the intermediate dosage induces flow cytometry artifacts, particularly in the cocktail-sensitive MNT-1 cells, whereas the ultra-low dosage induces neither artifacts nor changes in surface MHC expression in F0-1-β_2_m, SK-MEL 37, SK-MEL 93 and M10 (Fig. S1). The ultra-low dosage was therefore selected for further studies on activating NK cell ligands.

**Figure 5:**
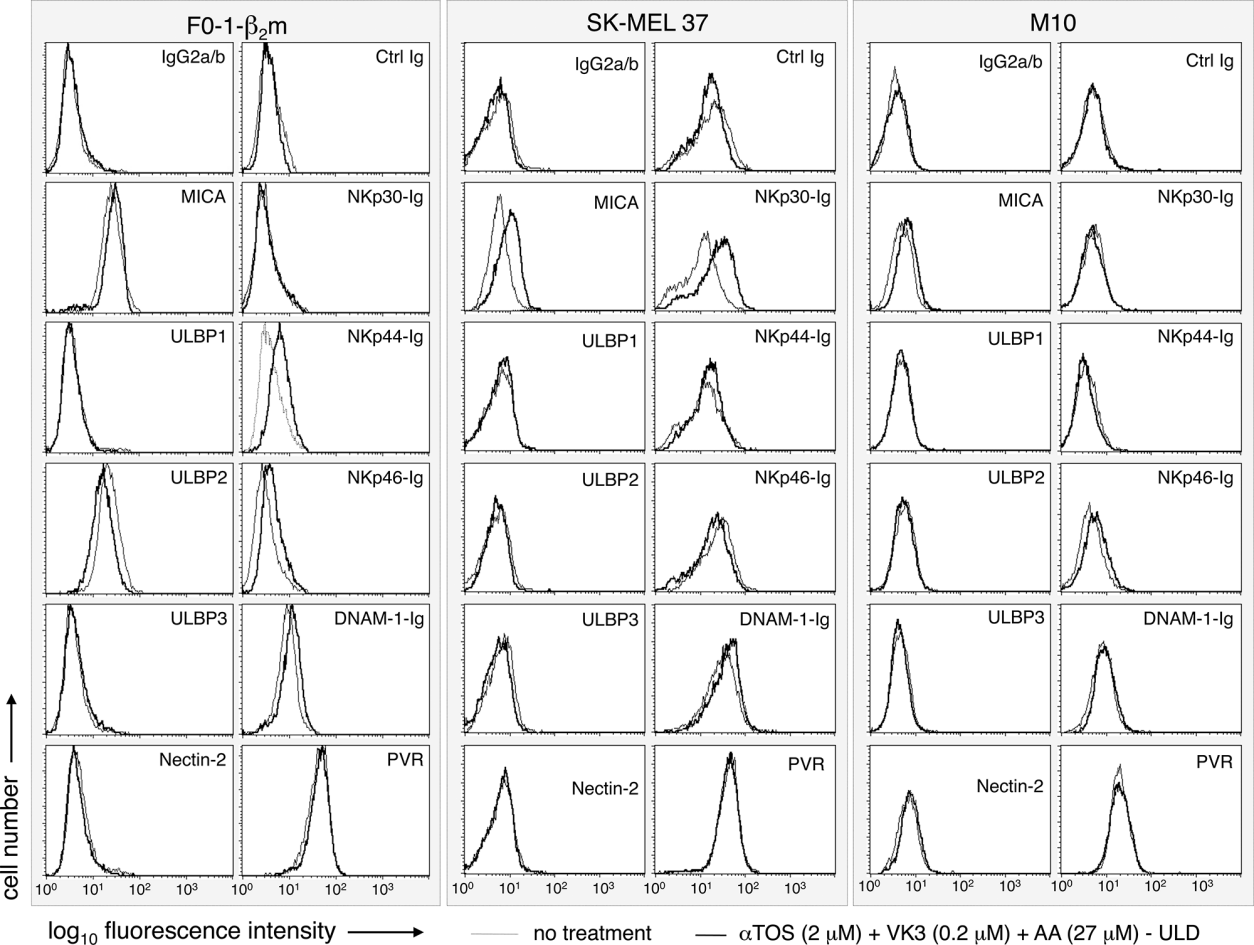
Effect of the αTOS/AA/VK3 cocktail on the surface expression of activating ligands of NK cells Melanoma cell lines were either left untreated, or treated for 16 h at the indicated concentrations of the cocktail, and tested by flow cytometry with mAbs or Ig fusion proteins, as indicated. An IgG2a/b isotype mix and an irrelevant Ig fusion protein were used as negative controls.

NKG2D ligands (MICA, ULBP-1, ULBP-2, and ULBP-3) and DNAM-1 ligands (Nectin-2 and PVR) were assessed by flow cytometry with specific mAbs. Ig fusion constructs were employed to cumulatively detect DNAM-1 ligands and the ligands of NKp30, NKp44, and NKp46. Treatment at the ultra-low dosage induced surface up-regulation in continuous F0-1-β_2_m, SK-MEL 37, and M10 cells in 3 separate experiments, one of which is shown in Fig. 5. MICA was variably up-regulated. Ligands for at least one of the NKp30, NKp44 and NKp46 receptors were up-regulated in F0-1-β_2_m and SK-MEL 37, but not in M10 cells. Occasionally, ULBP2 was very slightly decreased, whereas minimal or no effects were seen on ULBP1, ULBP3, Nectin-2 and PVR. Accordingly, binding of DNAM-1 Ig fusion proteins remained essentially unchanged, or was very slightly enhanced. Identical experiments with early-passage Mel 11 and Mel 24 cells demonstrated similar and strong up-regulation of either or both NKp44 and NKp46, and also revealed a slight down-regulation of MICA in Mel 24 only (Fig. S2). In contrast, SK-MEL 93 cells were reproducibly unresponsive (not shown).

It may be concluded that 5 out of 6 melanoma cell lines respond to the αTOS/AA/VK3 cocktail with moderate up-regulation, and occasionally slight down-regulation, of activating NK cell ligands. Use of VK3/AA combinations not containing αTOS had very similar effects as compared to the three-component cocktail in continuous cell lines (supplemental Table S1). Therefore, ultra-low dosages of AA/VK3 induce immunophenotypic up-regulation in melanoma cells resistant to the death-inducing effects of the cocktail, and the inclusion of αTOS does not detectably influence immunophenotype (Fig. 5), AIF translocation (Fig. 3C), or cell viability (Fig. 2).

### The αTOS/AA/VK3 cocktail enhances NK cell lysis of melanoma cells

**Figure 6:**
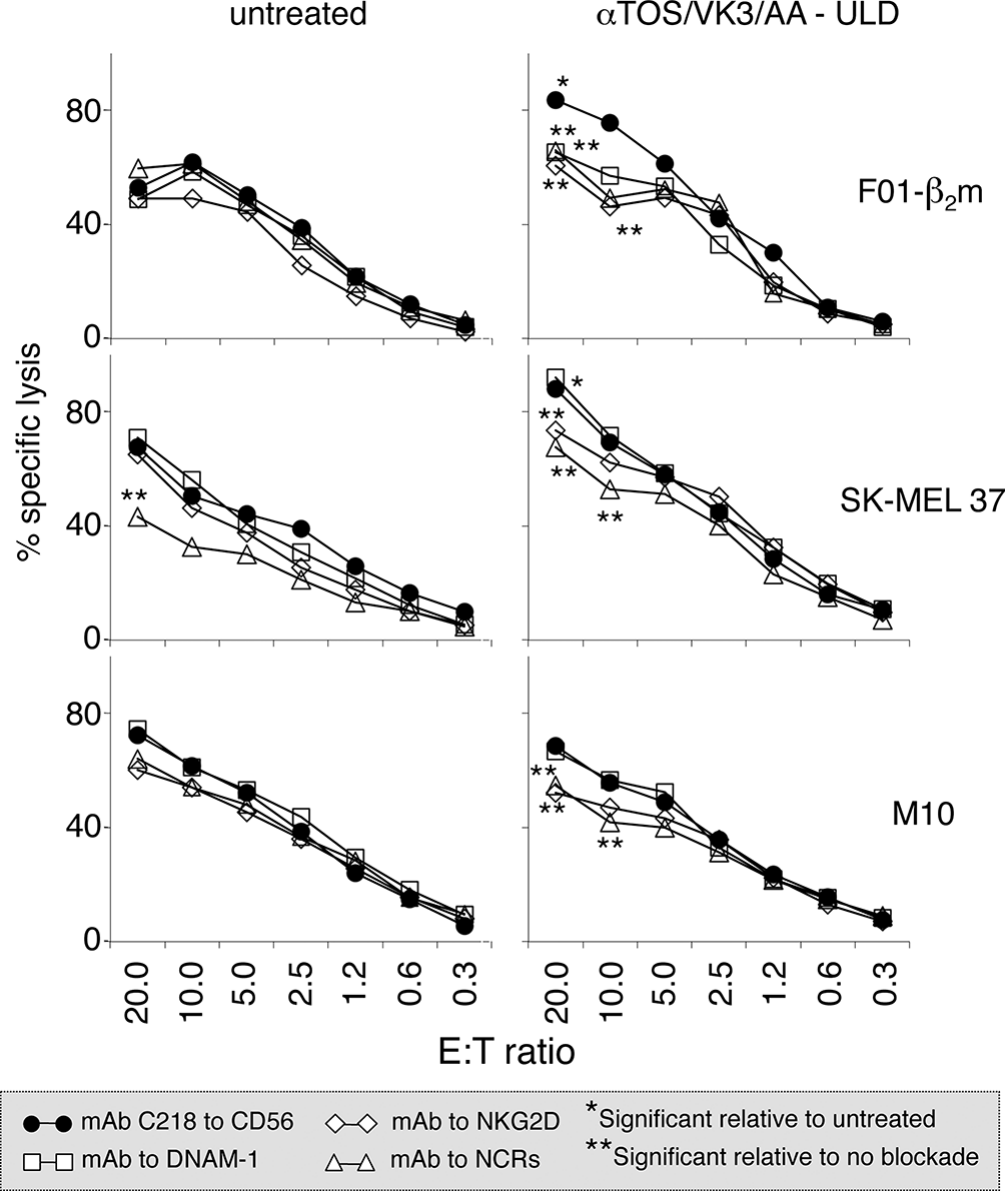
Effect of treatment with the αTOS/VK3/AA cocktail on the susceptibility of melanoma cells to lysis by NK cells Target melanoma cells treated with the cocktail at the ultra-low dosage for 16 h were compared to untreated cells in a standard ^51^Cr release assay, using polyclonal NK cells from a healthy donor as effectors, at the indicated Effector:Target (E:T) ratios. Effectors were pre-incubated either with a control mAb to CD56 that binds NK cells but does not significantly influence cytotoxicity [12, 14], or with blocking antibodies/Ig fusion proteins to DNAM-1, NKG2D, and Natural Cytotoxicity Receptors (NCRs; a mix of Ig fusions to NKp30, NKp44 and NKp46). Asterisks identify significant (*t*-test) differences (*p* < 0.04) at each E:T ratio, as follows. * cocktail-treated vs untreated target melanoma cells. ** DNAM-1, NKG2D, or NCR blockade vs CD56 control in the same graph.

To assess the functional consequences of the up-regulation of NK cell ligands, polyclonal NK cells (>98% CD56+/CD3-) were obtained by immunomagnetic sorting from the PBMCs of healthy donors in the absence of Interleukin-2 (IL-2). These effectors lyse less efficiently than *in vitro* IL-2-activated NK cells [14], providing a lytic readout that possibly more closely reflects physiological NK cell activity. Polyclonal NK cells were tested as effectors in microcytotoxicity assays using the F0-1-β_2_m, SK-MEL 37, SK-MEL 93 and M10 melanoma cell lines as targets. Early-passage Mel 11 and Mel 24 cells were not tested, since they non-specifically release ^51^Cr, presumably because these cell lines are poorly adapted to cell culture and become damaged upon detachment from plastic dishes. The αTOS/AA/VK3 cocktail enhanced the susceptibility to lysis of F0-1-β_2_m, SK-MEL 37 and M10, e.g. the three continuous cell lines in which NK cell ligands are up-regulated, and this effect was substantially blocked by antibodies or Ig fusion proteins to NKG2D and NCRs, as expected (Fig. 6). In contrast, lysis was not detectably enhanced in SK-MEL 93 cells (not shown) in which NK cell ligands are not detectably up-regulated. Thus, treatment with αTOS/VK3/AA at dosages insufficient to induce overt oxidative stress and cell death does enhance the susceptibility of melanoma cell lines to immune lysis by NK cells.

## DISCUSSION

Sub-lethal concentrations of VK3 and AA, together with αTOS at a sub-apoptotic dosage, have previously been shown to efficiently induce prostate carcinoma cell death as a result of DNA fragmentation, lysosomal/mitochondrial perturbation, and cytochrome c release, but in the absence of appreciable caspase activation [5, 13]. In the present study, we show that cell death/autoschizis may also be induced in melanoma cells at a standard low dosage, but with a major difference: in melanoma αTOS behaves as an antagonist of VK3/AA, since it inhibits nuclear translocation of the Apoptosis Inducing Factor (AIF).

Although this argues against the inclusion of αTOS in a death-inducing cocktail, at 20-fold cocktail dilutions (ultra-low dosage) αTOS no longer exerts antagonistic effects of any kind, whereas VK3 and AA concentrations are sufficient to up-regulate activating NK cell ligands. Thus, at least under the present experimental conditions, and with regard to the tested ligands, two- and three-component cocktails may be equally effective. The inclusion of αTOS, although unnecessary for immunophenotypic modulation, may be considered in view of its known favorable *in vivo* effects on the innate immune system [15, 16].

Of interest, all (4/4) the continuous melanoma cell lines, but only some (2/6) of the early/medium-passaged cell lines resisted death induction to an extent sufficient to reveal immunophenotypic up-regulation. In contrast, sub-liminal death-inducing dosages could not be identified in the 4 remaining early-/medium-passaged cell lines, presumably because limited adaption to growth in culture and/or other unknown factors exacerbated cocktail toxicity. Whatever the interpretation, in 6 resistant melanoma cell lines a similar threshold dosage was identified that does not depend on *in vitro* passaging: above and below this dosage, autoschizis and phenotypic up-regulation reveal the existence of two alternative but most likely integrated death pathways.

This is not surprising, since several immune functions have become integrated into general housekeeping functions during evolution. For instance, peptide antigens resulting from the proteasomal degradation of cellular proteins are re-cycled by the immune system as peptide antigens, and nascent proteins not destined for disposal are forcefully degraded to promptly alert the immune system for damaged, infected or transformed cells [17]. Likewise, the same promiscuous chaperones that act as monomers to assist in the folding of generic cellular glycoproteins have been incorporated in a single supramolecular complex, termed the peptide loading complex, that also contains MHC-I-specific chaperones [18, 19]. Integration between housekeeping functions and the immune system may also be exploited for therapy, as exemplified by bortezomid, a first-in-class proteasome inhibitor and anticancer drug that has become a standard treatment for myeloma primarily due to its ability to impair growth and survival while exerting a variety of less understood effects on the immune system [20].

Likewise, protection from cellular (oxidative) stress involves intracellular sensors inducing programmed cell death, as shown previously by many groups, and surface ligands alerting NK cells, as shown herein for the first time. As also shown herein, cell-autonomous death programs and extrinsic immune mechanisms operate at dosages differing from 100-fold to 20-fold, depending on the cell line. NK cells may thus be alerted much before a harmful toxic threshold is reached. Like in proteasomal degradation and chaperone-assisted peptide loading onto MHC molecules, immunity may be viewed as the ultimate, specialized add-on that complements generic self-defense mechanisms.

All the cocktail-responsive NK cell ligands tested herein (particularly MICA, but also the ligands of the NKp30, NKp44 and NKp46 receptors) signal stress to NK cells [6, 8, 21]. Application of oxidative stress taking advantage of relatively non-toxic natural compounds such as vitamin K, vitamin C, and an analogue of vitamin E, may have applicative interest. Other types of cellular stress (e.g. heat shock, ER stress response etc.) may be more difficult to induce, control and exploit.

Whereas activation appears to be the prevalent NK cell response to the αTOS/VK3/AA cocktail, in 2/6 cell lines we also observed down-regulation of activating ligands, particularly MICA in an early-passage cell line. This suggests caution, since self-inhibitory and negative feedback mechanisms may limit the potential benefit of direct NK cell activation. For instance, oxidative stress has been shown to up-regulate PVR on the surface of T lymphocytes, resulting in NK cell suppression of T cell responses [22]. Therefore, the response to oxidative stress is not one way, but dual, e.g. it is the result of integration between activation and inhibition. This is not surprising: the immune system has built-in internal balances, and NK cell activation is often mirrored by reduced T cell function, as recently reviewed by us [23]. Also of relevance, chronic exposure to activating ligands has long been known to tolerize NK cells [24], and lymphoid stress responses to activating NK ligands show high inter-individual variation and are tuned to individual dose bandwidths [25]. In spite of the above limitations, the αTOS/AA/VK3 cocktail employed herein has several favorable features, including an extremely low inherent toxicity (at ultra-low dosages), and the possibility of intermittent administration. These may help thwarting oxidative stress against the tumor, preventing chronic stimulation, and minimizing negative feedbacks.

Vitamins and vitamin analogues have been shown to sensitize tumor cells to chemotherapy [26]. If predominant NK cell activation will be confirmed by further studies, αTOS, VK3 and AA should be sufficiently safe to be used for intermittent chemoprevention and long-term/low-toxicity adjuvant (immuno)-therapy of melanoma.

## MATERIALS AND METHODS

### Cells and biochemicals

Characteristics and identity verification of continuous (F-01-β_2_m, M10, SK-MEL 37 and SK-MEL 93), early-passage (Mel 3, Mel 11, Mel 23, Mel 24, and Mel 35) and medium-term passaged (MNT-1) melanoma cell lines have been described [27, 28]. For further details on establishment, passaging and identity verification see Supplemental Materials and Methods. αTOS, VK3 and AA were obtained from Sigma (Sigma, St Louis, MO, USA). Genomic DNA was extracted by phenol/chlorophorm, and electrophoresed on a standard agarose (0.8%) gel.

### Cell viability and drug combination analysis

Melanoma cell lines were plated in 96-well flat-bottom tissue culture plates at 3.0 × 10^4^ per well, and allowed to attach overnight. Then, they were incubated for 24 hrs with αTOS (10–100 μM), VK3 (1–7 μM), and AA (100–2000 μM), alone or in combination. Cell viability was determined by the crystal violet assay (2% crystal violet in 2% ethanol). Absorbance was read at 570 nm in an ELISA plate reader, and control absorbance was normalized to 100%. Survival curves were generated and the IC_50_ values determined. The effect of single drugs and their combinations was assessed by plotting the percentage of dead cells after treatment, and by the combination index (CI), using the CalcuSyn1 software. CI is a quantitative measure of the degree of pharmacological interaction between different drugs. CI = 1.0 denotes additivity; CI > 1.0 denotes antagonism; CI values between 1.0 and 0.7, 0.7 and 0.3, and < 0.3 denote slight, moderate and strong synergism, respectively. Results are expressed as the mean ±SD of three independent experiments performed in eight replicate wells for each experimental point. Intracellular hydrogen peroxide levels were assessed using the fluorescent dye 2′7′-dichlorofluorescein diacetate (DCFDA; oxidized by hydrogen peroxide to DCF), as described in Supporting Information.

### Subcellular fractionation and western blotting

Cells were permeabilized and fractionated into cytosolic, organelle and nuclear fractions by permeabilization, centrifugation, and Dounce homogenization, as described in Supporting Information. The fractions were electrophoresed and blotted onto nitrocellulose filters for antibody staining.

### Flow cytometry

Melanoma cells were stained on ice with either fluorochrome-labeled antibodies, or with a predetermined, optimal (10 μg/ml) concentration of primary antibody/chimeric Ig. In the latter case, primary antibody binding was revealed by FITC-labeled rabbit antibodies to either mouse or human Ig (Dako, Glostrup, Denmark). Isotype-matched control antibodies, or a chimeric Ig of irrelevant specificity were included as negative controls. Specifically bound fluorescence was immediately analyzed without fixation by a FACScan flow cytometer (Beckton & Dickinson, Mountain View, CA). mAbs W6/32 [29] to MHC-I is secreted by a hybridoma obtained from ATCC. The pan class II HLA molecules mAb KUL-01 has been described [30]. Antibodies to MICA (159227), ULBP1 (170818), ULBP2 (165903), ULBP3 (166510), and recombinant human Fc chimeras bearing the binding site of immune receptors DNAM-1-Ig, NKp30-Ig, NKp44-Ig and NKp46-Ig, were from R&D Systems (Minneapolis, MN).

### NK cells and functional assays

Polyclonal NK cells (>98% CD3^−^/CD56^+^, as assessed by flow cytometry with antibodies UCHT1 and MOC-1 from Dako, Denmark) were established by culturing healthy donor PBMCs *in vitro* for 10 to 12 days on feeder layers of RPMI 8866 cells, as described [14]. Microcytotoxicity was measured by a standard 4 h ^51^Cr release assay, at the indicated E:T ratios, using as targets 5 × 10^3^ melanoma cells per microplate well in triplicate, as described [12]. Antibody SKII.4 to the PolyoVirus Receptor (PVR, CD155) from Dr. Marco Colonna, and antibodies to Nectin-2 (BD Pharmingen), NKp30, NKp44 and NKp46 (R&D Systems) were used in receptor-blockade experiments. NK cells were pre-incubated with antibodies and Ig fusion proteins (10 μg/ml) at room temperature for 15 min, and then dispensed into the 96 well microplates containing target cells for the ^51^Cr release assay.

### Statistical analysis

Comparisons among groups were determined by one-way ANOVA with Tukey *post-hoc* analysis. Differences with *p* < 0.05 were considered statistically significant. All the data generated in this study were analyzed using the SPSS software.

## SUPPLEMENTARY DATA FIGURES AND TABLE


