# Supplementary material for: Sub-apoptotic dosages of pro-oxidant vitamin cocktails sensitize human melanoma cells to NK cell lysis
Source: Oncotarget. 2015 Sep 5;6(31):31039–49. doi: 10.18632/oncotarget.5024 (PMC4741587; doi:10.18632/oncotarget.5024)
Supplement: Supplementary file 1 [file oncotarget-06-31039-s001.pdf]

SUPPLEMENTARY DATA

SUPPLEMENTAL RESULTS

Selection of the optimal dosages of the α TOS/AA/VK3 cocktail for immunophenotypic testing, and lack of effect on MHC class I and class II molecules

Melanoma cell lines were treated with the αTOS/AA/VK3 cocktail at the ultra-low dosage (ULD; 20-fold dilution of the standard low dosage), and at the intermediate dosage (ID; 15-fold dilution of the standard low dosage). Classical MHC class I (HLA-A, -B, -C in humans) and MHC class II (HLA-DR, -DQ, -DP) molecules were assessed by flow cytometry using mAb W6/32 [1] and mAb KUL-01 [2], respectively. Representative results in 4 melanoma cell lines demonstrate that: (a) at the ultra-low dosage there is no appreciable phenotypic modulation (Figure S1A); (b) at the intermediate, slightly cytotoxic dosage, cells gated by propidium iodide exclusion display sporadic changes in MHC-I and MHC-II expression (Figure S1B). However, these changes are artifactual, since they are: (i) different in different cells; (ii) poorly reproducible even in the same cell line upon repeated testing (at least three independent experiments for each cell line were performed, not shown); and (iii) associated with non-specific fluorescent antibody uptake even in the absence of specific primary mAb (panel B, see Ig isotype controls). All these artifacts are particularly evident in MNT-1 cells, that are electively sensitive to the toxic effects of the cocktail. We conclude that changes in MHC class I and MHC class II expression occurring at $1/15^{th}$ of the standard low dosage depend most likely on toxic disruption of cell permeability and membrane integrity in dying cells, and are therefore to be regarded as non-specific. Thus, MHC antigens are not modulated by the αTOS/VK3/AA cocktail.

Immunophenotypic up-regulation of activating NK cell ligands in early-passage Mel 11 and Mel 24 melanoma cell lines

Mel 11 and Mel 24 were treated with the three-component vitamin cocktail in three separate experiments exactly as described for long-term passaged melanoma cell lines. Flow cytometry was carried out with the full panel of antibodies and Ig fusion constructs. Figure S2 depicts representative results of reproducible (3/3 experiments) up-regulation. Treatment resulted in increased binding of NKp44 and NKp46 Ig fusion proteins, and decreased binding of an antibody to MICA.

SUPPLEMENTARY METHODS

Cell lines

Melanoma cell lines were grown in RPMI 1640 medium supplemented with L-glutamine (2 mM) and 10% FBS. Long-term passaged, continuous cell lines F0–1-β_2m, M10, SK-MEL 37 and SK-MEL 93 were obtained from the originators and are described [3]. The highly differentiated, pigmented MNT-1 cell line was established by us [4] but has been carried in culture for >50 passages. Mel 3, Mel 11, Mel 23, Mel 24, and Mel 35 belong to another large series of early-passage (<20 passages) melanoma cell lines established by us [5, 6]. All these cells are routinely verified in our laboratory by HLA typing, that is a very effective measure to detect cross-contamination of cell cultures, as described [7].

Assessment of intracellular hydrogen peroxide

Intracellular hydrogen peroxide levels were assessed using the fluorescent dye 2′7′-dichlorofluorescein diacetate (DCFDA; oxidized by hydrogen peroxide to DCF). F0–1-β_2m cells (3×10^4) were seeded in 96-well plates, supplemented with 20 µM DCFDA per well, and treated with pro-oxidants. After treatment, the fluorescent probe was removed and the cells were washed, resuspended in PBS, and analyzed by a fluorescence plate reader (Infinite F200 PRO, Sunrise, Tecan, Männedorf, Swiss). The excitation and emission filters were set at 485 nm and 530 nm, respectively. The levels of Reactive Oxygen Species (ROS) were expressed as percentage increases in fluorescence calculated by the formula $[(Ft_x - Ft_0)/Ft_0 * 100]$, where Ft_x is fluorescence at the time of treatment, and Ft_0 is fluorescence at time 0.

Subcellular fractionation and Western Blotting

F0–1-β_2m cells (3×10^5 per well in 6-well plates) were treated with αTOS, VK3 and AA for 180 min, harvested, and the pellet was re-suspended in a cell permeabilization buffer containing digitonin (Trevigen, Gaithersburg, MD). The supernatant containing the cytosolic fraction was collected. The remaining pellet (organelle fraction) was lysed in RIPA buffer (20 mM Tris-HCl, pH 7.5, 150 mM NaCl, 1 mM Na_2EDTA, 1 mM EGTA, 1% NP-40, 1% sodium deoxycholate, 2.5 mM sodium pyrophosphate, 1 mM β-glycerophosphate, 1 mM Na_3VO_4, and 1 µg/ml protease inhibitors). To prepare the nuclear fraction, the cells were washed twice with ice-cold PBS and re-suspended in PBS

containing 10 mM $NaPO_3$, 150 mM NaCl, 0.5% Triton X-100, pH 7.4, supplemented with protease inhibitors. After 20 strokes in a Dounce homogenizer, the unbroken cells were spun down at 2,500 × g for 15 min at 4°C. The nuclear fraction was re-suspended in ice-cold hypertonic buffer (10 mM $NaPO_3$, 350 mM NaCl, pH 7.4), mixed and sonicated on ice (5 × 20 s). After centrifugation at 2,500 × g for 15 min at 4°C, the supernatant was collected. For western blot analysis, protein samples (50 µg per lane) were resolved using 12.5% SDS-PAGE, transferred to nitrocellulose membranes, and incubated overnight with anti-AIF (Cell Signaling Technology). β-Actin and laminin (Bethyl, Montgomery, TX, USA) were used as loading controls for the cytosolic and nuclear fractions, respectively. Following incubation with an Horse Radish Peroxidase conjugated secondary IgG (Sigma), the blots were developed using the ECL detection system (Pierce Biotechnology, Rockford, IL, USA). Band intensities were visualized by ChemiDoc using the Quantity One software (BioRad).

REFERENCES

1. Parham P, Barnstable CJ, Bodmer WF. Use of monoclonal antibody (W6/32) in structural studies of HLA-A,B,C antigens. J Immunol. 1979; 23:342–349.
2. Giacomini P, Tecce R, Nicotra MR, Cohen BB, Mazzilli MC, Natali PG. mAb KUL/05 identifies a denaturation-resistant determinant shared by class II MHC products DR, DQ and DP. J Immunogen. 1989; 16:203–216.
3. Fruci D, Ferracuti S, Limongi MZ, Cunsolo V, Giorda E, Fraioli R, Sibilio L, Carroll O, Hattori A, van Endert PM, Giacomini P. Expression of endoplasmic reticulum aminopeptidases in EBV-B cell lines from healthy donors and in leukemia/lymphoma, carcinoma, and melanoma cell lines. J Immunol. 2006; 176:4869–4879.
4. Giacomini P, Fraioli R, Cuomo M, Natali PG. Membrane compartmentalization of melanosomal gp75. J Invest Dermatol. 1992; 98:340–342.
5. Giacomini P, Giorda E, Fraioli R, Nicotra MR, Vitale N, Setini A, Delfino L, Morabito A, Benevolo M, Venturo I, Mottolese M, Ferrara GB, Natali PG. Low prevalence of selective human leukocyte antigen (HLA)-A and HLA-B epitope losses in early-passage tumor cell lines. Cancer Res. 1999; 59:2657–2667.
6. Giorda E, Sibilio L, Martayan A, Moretti S, Venturo I, Mottolese M, Ferrara GB, Cappellacci S, Eibenschutz L, Catricalà C, Grammatico P, Giacomini P. The antigen processing machinery of Human Leukocyte Antigens: linked patterns of gene expression in neoplastic cells. Cancer Res. 2003; 63:4119–4127.
7. Giacomini P, Giorda E, Pera C, Ferrara GB. An ID card for tumour cell lines: HLA typing can help. Lancet Oncol. 2001; 2:658.

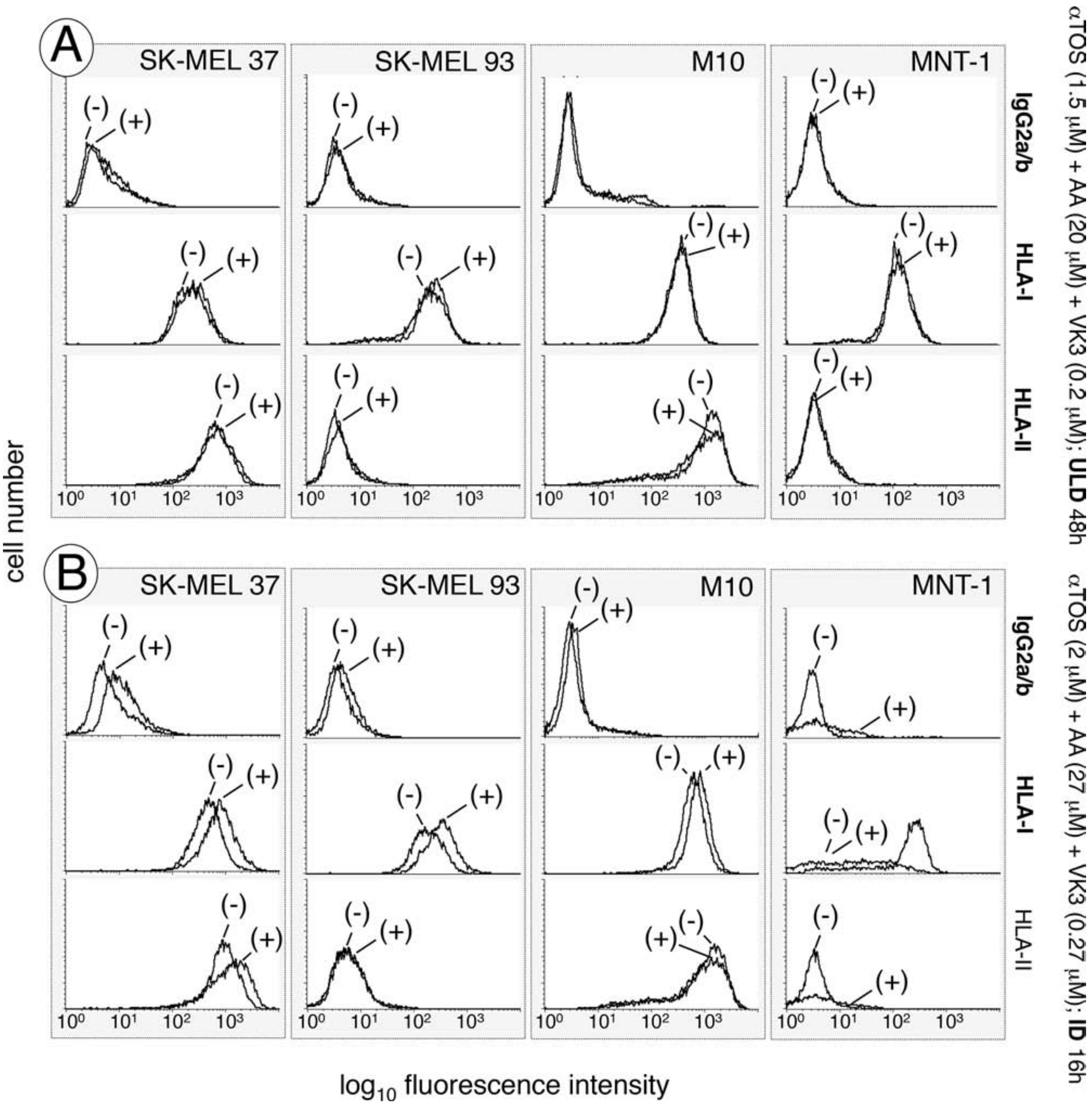

Supplementary Figure S1: Effect of the αTOS/AA/VK3 cocktail on the surface expression of MHC class I and MHC class II molecules. The 4 indicated melanoma cell lines were grown in the absence (-) and presence (+) of the aTOS/VK3/AA cocktail at two different concentrations and for different times (A and B). Then, they were tested by flow cytometry with an IgG2a/b isotype control, mAb W6/32 to HLA class I, and mAb KUL/01 to HLAclass II molecules.

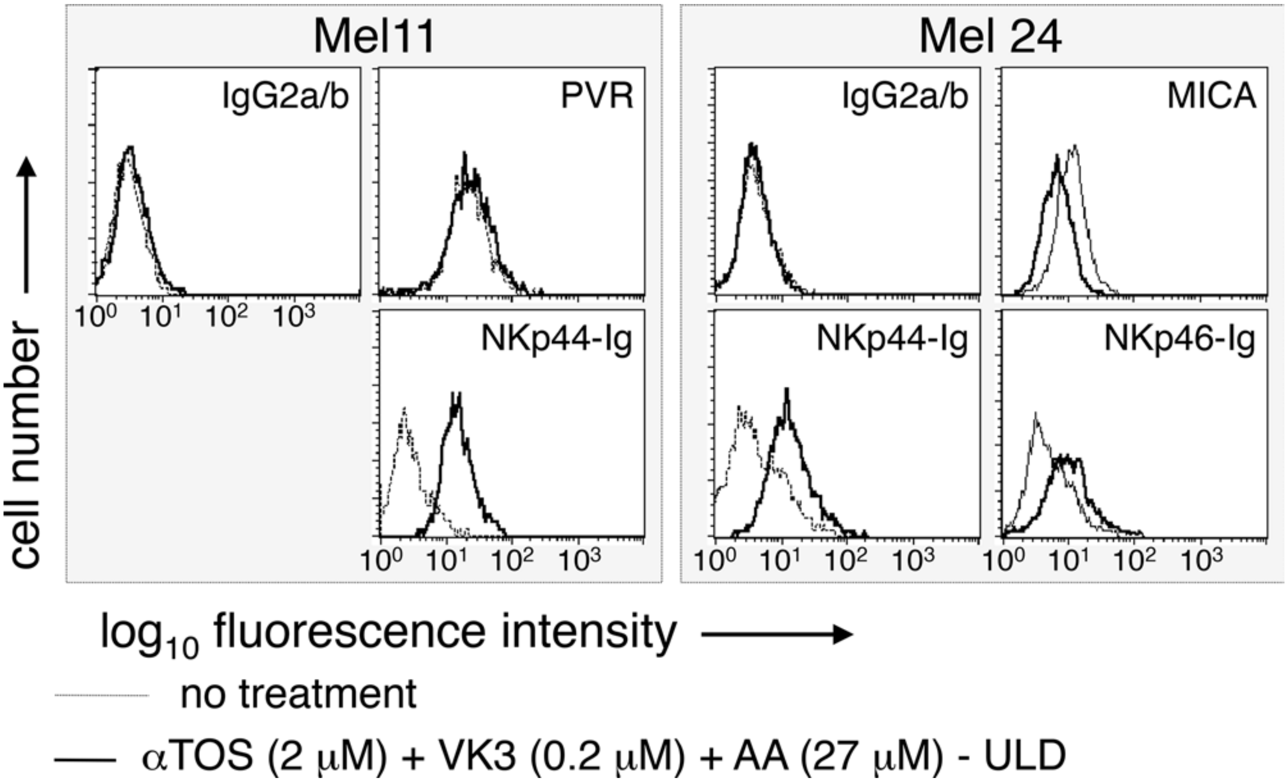

Supplementary Figure S2: Immunophenotypic up-regulation of early-passage melanoma cell lines. The indicated cell lines were either left untreated, or treated for 16 h at the indicated concentrations of the cocktail, and tested by flow cytometry with mAbs or Ig fusion proteins, as indicated. An IgG2a/b isotype mix (upper left in both panels) and an irrelevant Ig fusion protein (not shown) were used as negative controls. Only selected histograms are shown of representative mAbs and Ig fusion proteins. ULD, Ultra-Low Dosage.

Supplementary Table S1: Effect of pro-oxidant cocktails on the expression of activating immune ligands: a comparison between two-component and three-component formulations[1]

	F0-1-β_2m		
	control	VK3 + AA	VK3 + AA + αTOS
IgG1/2	4[2]	5	4
MICA	34	**42**	**48**
ULBP1	7	5	4
ULBP2	14	12	**7**
ULBP3	5	5	7
Nectin-2	10	7	6
PVR	85	90	75
IgG chimera	7	8	8
DNAM-1-Ig	9	**23**	**13**
NKp30-Ig	7	7	6
NKp44-Ig	7	**12**	**13**
NKp46-Ig	7	**12**	**14**

[1] 18 h reatment with pro-oxidant cocktails both containing Vit K3 0.2 µM and AA20 µM.When presentαTOS was provided at 1.5 µM.

[2] Mean fluorescence intensity. Immunophenotypic changes are boldface.
